# Novel Mouse Model Reveals Distinct Activity-Dependent and –Independent Contributions to Synapse Development

**DOI:** 10.1371/journal.pone.0016469

**Published:** 2011-01-31

**Authors:** Pier Giorgio Pacifici, Christoph Peter, Pessah Yampolsky, Michael Koenen, Joseph J. McArdle, Veit Witzemann

**Affiliations:** 1 Department of Molecular Neurobiology, Max Planck Institute for Medical Research, Heidelberg, Germany; 2 Department of Pharmacology and Physiology, New Jersey Medical School, Newark, New Jersey, United States of America; Medical College of Georgia, United States of America

## Abstract

The balanced action of both pre- and postsynaptic organizers regulates the formation of neuromuscular junctions (NMJ). The precise mechanisms that control the regional specialization of acetylcholine receptor (AChR) aggregation, guide ingrowing axons and contribute to correct synaptic patterning are unknown. Synaptic activity is of central importance and to understand synaptogenesis, it is necessary to distinguish between activity-dependent and activity-independent processes. By engineering a mutated fetal AChR subunit, we used homologous recombination to develop a mouse line that expresses AChR with massively reduced open probability during embryonic development. Through histological and immunochemical methods as well as electrophysiological techniques, we observed that endplate anatomy and distribution are severely aberrant and innervation patterns are completely disrupted. Nonetheless, in the absence of activity AChRs form postsynaptic specializations attracting motor axons and permitting generation of multiple nerve/muscle contacts on individual fibers. This process is not restricted to a specialized central zone of the diaphragm and proceeds throughout embryonic development. Phenotypes can be attributed to separate activity-dependent and -independent pathways. The correct patterning of synaptic connections, prevention of multiple contacts and control of nerve growth require AChR-mediated activity. In contrast, myotube survival and acetylcholine-mediated dispersal of AChRs are maintained even in the absence of AChR-mediated activity. Because mouse models in which acetylcholine is entirely absent do not display similar effects, we conclude that acetylcholine binding to the AChR initiates activity-dependent and activity-independent pathways whereby the AChR modulates formation of the NMJ.

## Introduction

The vertebrate neuromuscular junction (NMJ) is a large cholinergic synapse where transmission occurs through acetylcholine (ACh). However, it is unclear how the interaction of activity-dependent and -independent factors coordinates the differentiation of synaptic elements to achieve the precise development of NMJs. The present study tests the hypothesis that the muscle ACh receptor (AChR) is central to this process.

Chronologically, embryonic AChRs composed of α_2_βγδ subunits (AChRγ) cluster on skeletal muscle fibers through prepatterning, a nerve-independent process requiring the muscle-specific tyrosine kinase MuSK [1], and a growing number of additional proteins. Subsequently, nerve-derived factors stabilize AChR clusters on the muscle membrane [2], [4], [5], [6]. Evidence suggests that ACh initiates dispersion of aneural AChR aggregates [1], [7], [8]. During later embryonic stages and early postnatal development, adult AChRs composed of α_2_βεδ subunits (AChRε) replace AChRγ [9]. The mechanisms determining NMJ positioning and the signals stopping axonal branching to form correctly located stable synaptic contacts remain unknown.

It is therefore important to thoroughly evaluate the role of AChR activity as a mechanism for regulating formation, maintenance and development of the NMJ. However, knock-out lines incur in a resolution problem: they cannot distinguish between phenotypes caused by the absence of synaptic transmission, and phenotypes arising from the disruption of molecular signaling pathways caused by the absence of crucial molecules such as ACh or AChR, which may lead to non-physiologically relevant alterations. Furthermore, electrophysiological activity-independent effects of ACh could not be detected with previous *in vivo* studies. Therefore, one needs a unique mouse model to distinguish between activity-dependent and activity-independent roles of ACh.

In humans, genetic defects arising from mutations of genes coding for subunits of the AChR cause congenital myasthenic syndromes [10]. One mutation, leading to a fast-channel syndrome, is located on the ε subunit and greatly decreases the rate of channel opening, reducing the affinity for ACh [11]. This mutation was used to generate *γ/ε-fc* mice, in which a mutated ε subunit cDNA fragment is fused into the γ subunit gene by homologous recombination to replace AChRγ with functionally “silent” receptors (AChRγ/ε-fc) during embryonic development.

Our findings show that AChRγ/ε-fc is expressed, clustered in endplate-like structures, and developmentally regulated like AChRγ in wild type mice (WT). Lack of AChR-mediated activity, however, leads to a series of synaptic abnormalities during embryonic development, which are not rescued at later developmental stages when AChRε starts replacing the AChRγ/ε-fc. The *γ/ε-fc* mice exhibit abnormal endplate distribution, axonal growth, muscle innervation, NMJ formation and distribution, as well as endplate morphology. However, the *γ/ε-fc* mice differ from phenotypes reported for mouse lines whose motor nerves do not release ACh [12], [13], [14]. Thus, the *γ/ε-fc* mouse line demonstrates the contribution of activity-dependent and -independent AChR-mediated processes to the formation of NMJs.

## Results

### Directed genetic impairment of postsynaptic activity by targeting the AChR γ subunit gene

**Figure 1:**
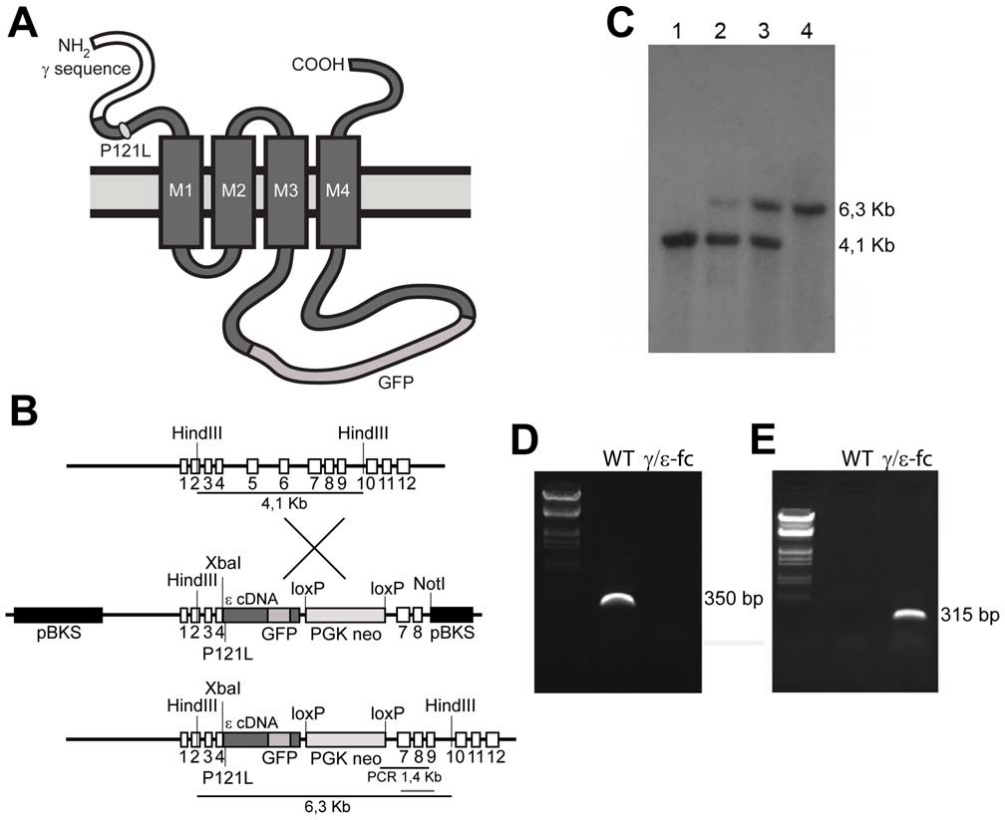
Generation and Structure of the *γ/ε-fc* Gene. (A) Schematic representation of the recombinant AChRγ/ε-fc subunit, highlighting the location of the P121L mutation and the GFP insertion into the large cytoplasmic loop. (B) Diagram of the wild type γ subunit and targeting vector structure. Coding DNA sequences are represented by boxes. The ε cDNA (dark gray box) includes the P121L mutation and humanized EGFP sequence in the large cytoplasmic loop and is inserted into the genomic γ subunit DNA sequence at a XbaI restriction site in exon 4. The PGKneo cassette is spliced out of the primary mutated AChR ε subunit transcript. (C) Southern blot analysis of DNA from a wild type mouse (lane 1), positive ES cell clone (lane 2), heterozygous mouse (lane 3) and homozygous mouse (lane 4). The wild type γ subunit is represented by a 4.1 Kb fragment; the AChRγ/ε-fc subunit is represented by a 6.3 Kb fragment as indicated in B. (D, E) RT-PCR on RNA extracted from muscle of wild type (WT) or *γ/ε-fc* mice (γ/ε-fc). The γ subunit-specific primers yield a 350 bp fragment only for RNA from wild type muscles (D). The AChRγ/ε-fc subunit-specific primers yield a 315 bp DNA fragment for RNA from muscles of homozygous *γ/ε-fc* animals (E).

In human patients, a fast channel myasthenic syndrome is caused by a P121L mutation in the AChRε subunit, reducing the postsynaptic response to ACh while retaining a normal AChR load on the endplates (Ohno et al, 1996). The same mutation in recombinant mouse AChR expressed in *X. laevis* oocytes caused a similar dramatic reduction in ion conductance [15]. Therefore, we used homologous recombination to replace the WT γ subunit with a construct comprised of a fusion γ/ε subunit (*γ/ε-fc* subunit) that carried the P121L mutation. GFP was inserted for direct visualization of mutant receptors, AChRγ/ε-fc, that are expressed under the control of the native γ subunit promoter. The structurally intact AChRs are expected to be functionally “silent” under physiological conditions (Fig. 1A, B; see Material and Methods). Successful homologous recombination of the *γ/ε-fc* construct in embryonic stem cells was confirmed by Southern blot (Fig. 1C) and RT-PCR (Fig. 1D–E).

**Figure 2:**
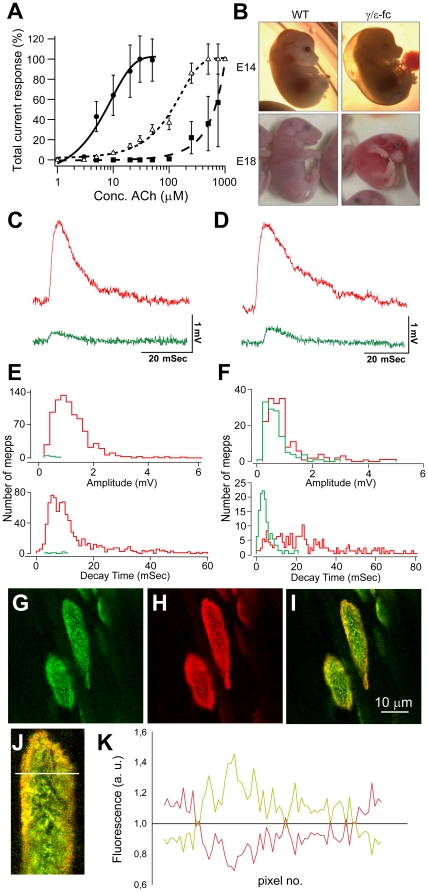
Electrophysiological and molecular characterization of the *γ/ε-fc* mice. (A) Whole cell current responses of *Xenopus laevis* oocytes expressing recombinant AChRε (•) or mutant AChR containing γP121L (Δ) and εP121L (▪) subunits at different concentrations of ACh (experimental details see Peter et al., 2005). Data are represented as mean (n = 9–11) ± SEM. (B) *γ/ε-fc* embryos and their wild type (WT) littermates at embryonic day 14 (E14) and 18 (E18). (C) Sample miniature endplate potentials (mepps) recorded in diaphragm muscles of E16 and (D) E18 embryos having the WT (red) and *γ/ε-fc* (green) genotype. (E) Distributions of amplitude and 90% to 10% decay times for mepps recorded from E16 and (F) E18 embryos. Red bars indicate WT and green bars indicate *γ/ε-fc*. The numbers of mepps, endplates, and muscles providing the data for each histogram were as follows. E16 embryos: 831, 29, 3 (WT) and 6, 72, 5 (*γ/ε-fc*); E18 embryos 170, 45, 3 (WT) and 123, 42, 6 (*γ/ε-fc*). (G–I) Confocal images of *γ/ε-fc* endplates in an E19 diaphragm: (G) GFP fluorescence present only in receptors containing the AChRγ/ε-fc subunit, (H) R-bgt staining of the full AChR load of AChRγ/ε-fc and AChRε, (I) Overlay showing accumulation of adult AChRε on the edges of the endplate. Scale bar: 10 µm. (J) Detail of the above endplate; the white line indicates a sample measurement area (see Material and Methods). (K) Fluorescence intensity measurement illustrates differential distribution of GFP-tagged AChRγ/ε-fc and adult AChRε. The green line represents green to red fluorescent intensity ratios averaged along 5 transversal sections as indicated in (J) being smallest at the edges of the endplate. The red line represents the ratio of red to green fluorescence which is highest at the edges where AChRε are inserted.

Fig. 2A shows dose-response curves of recombinant AChRs in *X. laevis* oocytes that were analysed as described by Peter et al. (2005). The results show that ACh binding affinities in AChR containing γ subunits with the P121L mutation (AChRγ_P121L_) are significantly reduced compared to WT AChR. The reduction is much stronger, however, in mutated ε subunits (AChRε_P121L_). Therefore, we used a γ/ε fusion construct to generate “silent” AChRγ/ε-fc. The heterozygous *γ/ε-fc* mice are viable and indistinguishable from WT littermates. In contrast, homozygous *γ/ε-fc* embryos have abnormally pigmented skin and are reduced in size, present a subcutaneous cavity on the dorsal side and assume a curled posture (Fig. 2B). These homozygous embryos fail to exhibit spontaneous movement and die at birth, presumably due to respiratory paralysis.

Evaluation of miniature endplate potentials (mepp) of E16 and E18 *γ/ε-fc* embryos (Fig. 2 C to F) suggests, indeed, that the AChRγ/ε-fcs are not functional. Microelectrodes were inserted into muscle fibers of eight E16 WT embryos. While 45% of these control impalements detect mepps, the success rate for mepp detection is 4% for five E16 *γ/ε-fc* diaphragms (see Supplementary Table S1). Furthermore, mepps are lower in frequency at the endplates of *γ/ε-fc* embryos (mean mepp frequency is 0.03±0.01 Sec^−1^ for 3 *γ/ε-fc* endplates compared to 0.39±0.20 Sec^−1^ for the 37 WT endplates). As summarized in Figure 2E, mepp amplitude and 90% to 10% decay time are also reduced for *γ/ε-fc* mepps. The mean values for mepp amplitude and decay time for WT preparations are 1.18±0.03 mV and 13.5±0.46 mSec (829 mepps, 37 endplates, 3 embryos). These values are significantly (p<0.01) greater than the corresponding *γ/ε-fc* values of 0.38±0.08 mV and 7.3±1.12 mSec (6, 3, 3). In *γ/ε-fc* E18 embryos, the percentage of impalements detecting mepps increases to 26%. However, mepp detection in WT impalements remains greater (64%). Furthermore, the mean mepp amplitude and decay time values of 0.68±0.04 mV and 4.72±0.31 mSec (123, 42, 5) are significantly (p<0.01) less than the corresponding WT values of 1.01±0.06 mV and 25.0±1.36 mSec (170, 47, 3) (see Fig. 2F). This analysis suggests that functional endplates are far less frequent for *γ/ε-fc* embryos. Low expression of the adult AChRε, as indicated by the shorter decay time of the mepps of *γ/ε-fc* preparations, would account for the smaller mepps observed at a lower mean frequency at endplates of mutant mice.

AChRγ/ε-fc in homozygous animals is assembled and shuttled to the membrane as clearly demonstrated by the presence of GFP fluorescence in endplates of *γ/ε-fc* embryos. AChRγ/ε-fcs bind rhodamine-labeled α-bungarotoxin (r-bgt), revealing co-localized GFP and r-bgt fluorescence in *γ/ε-fc* endplates (Fig. 2G–I). In E19 *γ/ε-fc* endplates AChRε is inserted at peripheral regions as clearly detected by r-bgt staining (Fig. 2 G–K). This occurs earlier than in WT animals, probably due to highly reduced (<50%) *γ/ε-fc* subunit transcript levels in comparison to γ in WT mice (Pacifici, Doctoral thesis, University of Heidelberg, 2009). A similar premature and directed incorporation of AChRε has been observed in mice expressing γ-GFP fusion subunits [16].

Together, these observations suggest that green fluorescent AChRγ/ε-fc channels are structurally intact. They bind α-bungarotoxin with high affinity, replace the WT AChRγ channels in *γ/ε-fc* endplates, and their expression is under proper developmental control. Measurement of acetylcholine-induced mepps, however, indicates that the AChRγ/ε-fc channels are indeed not functional.

### Anatomy of the Diaphragm NMJ

Replacement of AChRγ by AChRγ/ε-fc raises fundamental questions concerning the influence of the modified AChRs on early endplate differentiation. For example, does the *γ/ε-fc* mutation modify the nerve-independent prepatterning process, nerve/muscle contact formation and stabilization, the dispersion of non-synaptic AChR aggregates, and the overall development of muscle fibers?

**Figure 3:**
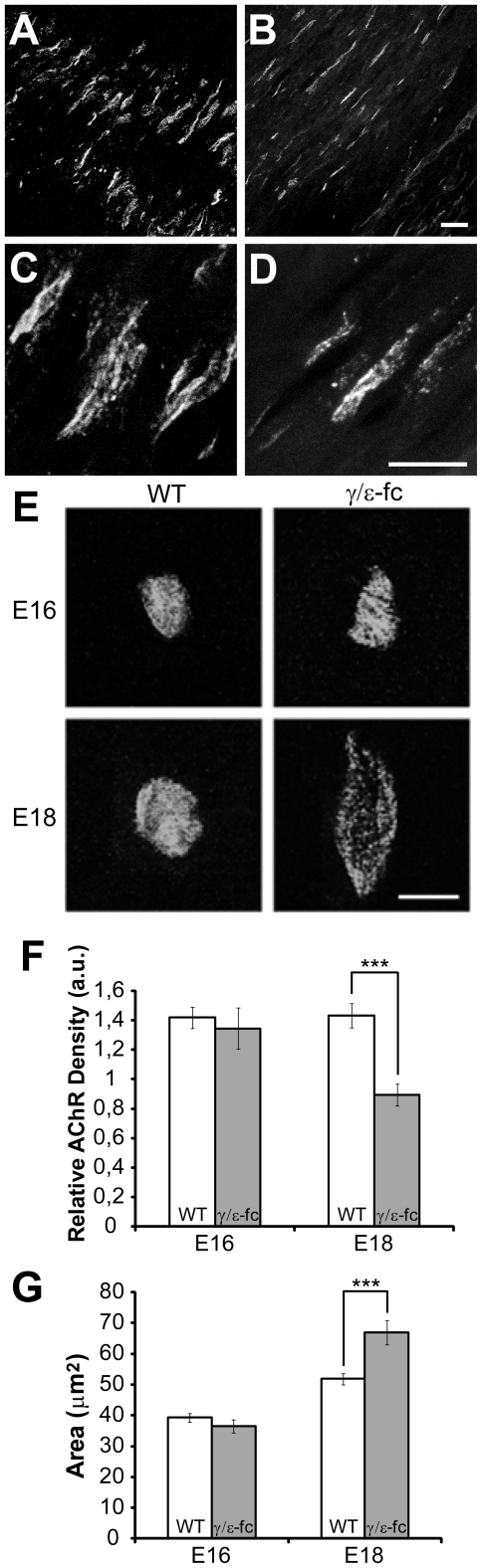
Endplate morphology. (A–D) Confocal images of prepatterned receptors in diaphragm of E14 embryos, visualized with Alexa-488-labeled α-bungarotoxin. (A) WT endplates and (B) *γ/ε-fc* endplates. AChRγ/ε-fc clusters are spread over a wider region and appear thinner than WT AChRγ (scale bar: 10 µm). (C, D) Higher resolution images (scale bar: 20 µm). (C) WT and (D) γ/ε-fc clusters have similar receptor densities. (E) Confocal images of single WT and *γ/ε-fc* endplates of E16 and E18 diaphragms visualized with Alexa-488-labeled α-bungarotoxin. E16 WT and *γ/ε-fc* endplates reveal no gross morphological differences. At E18, the WT endplate and the *γ/ε-fc* show morphological difference (scale bar: 10 µm). (F) Relative receptor densities measured as fluorescence intensities (see Material and Methods) are given in arbitrary units (a.u.). WT (white) and *γ/ε-fc* (gray) endplates at E16 and E18 (n = 60 endplates, 3 embryos per point). All data are represented as mean ± SEM). (G) Area (in µm^2^) of WT (white) and *γ/ε-fc* (gray) endplates at E16 and E18. n = 60 endplates, 3 embryos per point. All data are represented as mean ± SEM. (See also Figure S1).

At E14, AChRs in the *γ/ε-fc* diaphragm are clustered similarly to WT. However, AChRγ/ε-fc clusters appear thinner and more widespread than in WT (Fig. 3A–D). At E16 similar endplate structures have evolved in WT and *γ/ε-fc* diaphragm (Fig. 3E). At later developmental stages, the endplates of *γ/ε-fc* embryos present elongated, irregular shapes and unevenly clustered AChRγ/ε-fcs appear speckled in comparison to WT receptors (Fig. 3E).

The average AChR density (see Fig. 3F) in E16 *γ/ε-fc* embryos (1,43±0,08 a.u.) is similar to WT (1,41±0,07 a.u.). However, at E18, the AChR density in *γ/ε-fc* endplates is significantly reduced (0,89±0,07 a.u.) compared to that of WT littermates (1,34±0,14 a.u.) as well as earlier developmental stages. The average endplate area of E16 *γ/ε-fc* embryos (36,53±2,08 µm^2^) is equivalent to that of their WT littermates (39,23±1,43 µm^2^). At E18, however, the endplate area of *γ/ε-fc* animals (66,92±3,98 µm^2^) is significantly larger than that of WT littermates (51,83±1,90 µm^2^) (Fig. 3G). Expressing AChR density relative to endplate area indicates that the overall number of receptors for E18 *γ/ε-fc* embryos is reduced by less than 20% of WT suggesting that the γ/ε-fc subunit has only a minimal effect on AChR membrane assembly. Therefore, the developmental increase of differences in endplate anatomy may reflect the increasing importance of postsynaptic activity in NMJ maturation.

### Endplates are scattered in the diaphragms of *γ/ε-fc* embryos

**Figure 4:**
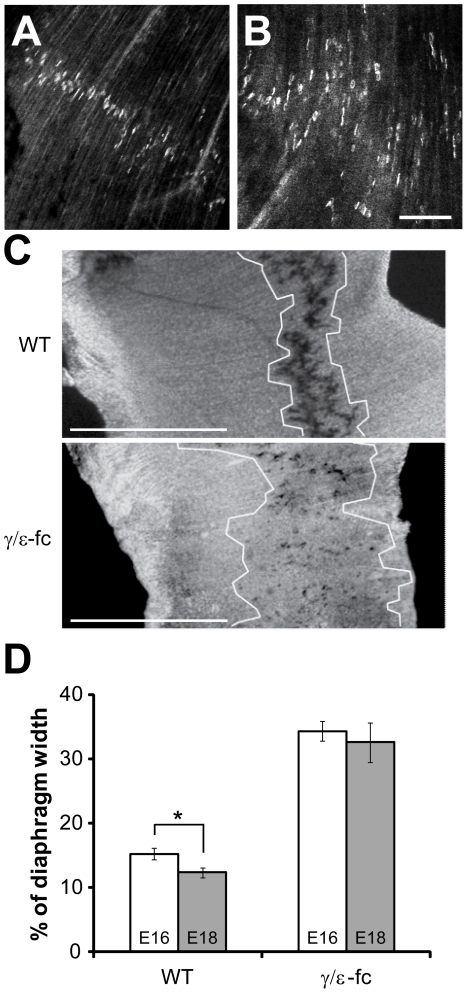
Endplate distribution. (A) Confocal images of endplate distribution in WT diaphragm of E18 embryos, visualized with Alexa-488-labeled α-bungarotoxin and (B) *γ/ε-fc* homozygous embryos. Scale bar: 100 µm. (C) E18 diaphragms from wild type (WT) and *γ/ε-fc* homozygous embryos stained histochemically for AChE enzyme activity. The width of the endplate band was measured as detailed in Material and Methods and is marked schematically by white boundaries. Scale bar: 1 mm. (B) Comparison of total endplate distribution widths (as percentages of total diaphragm width) between embryos within the same group (WT, white; or *γ/ε-fc*, gray) at ages E16 and E18. n = 27 measurements, 2 embryos per point. All data are represented as mean ± SEM.

The endplates in *γ/ε-fc* diaphragms are not located centrally as in WT diaphragms (Fig. 4A) but are spread out across the diaphragm (Fig. 4B). Since acetylcholinesterase (AChE) clusters have been shown to identify synaptic sites reliably in *ChAT^−/−^* mice [12], [14] we stained *γ/ε-fc* diaphragms for AChE activity [17]. The distribution of endplates is dramatically different from that observed for WT embryos and even at late embryonic stages the endplate band of *γ/ε-fc* diaphragms is dispersed over the width of the diaphragm (Fig. 4C; Table 1). In WT, the percentage of diaphragm width occupied by the endplate band (%DW) in E18 embryos is reduced when compared to E16 embryos (p = 0,022), due to the lateral growth of myotubes, which increases diaphragm width without affecting endplate distribution. In contrast, the %DW of E18 and E16 *γ/ε-fc* embryos does not significantly change (p>0,6). This implies that the absolute width of the endplate band increases for *γ/ε-fc* mice because new endplates are continuously formed in myotubes even at late developmental stages. This phenotype of the *γ/ε-fc* mutant is unique as opposed to that of *ChAT^−/−^* mice, where the absolute endplate band width remains constant through late developmental stages.

**Table 1:** Endplate band width as percentage of total hemidiaphragm width (%DW).

*Sample calculations of %DW*						
	Wild type			γ/ε-fc		
	*Diaphragm width (DW) (µm)*	*Endplate band width (µm)*	*%DW*	*Diaphragm width (DW) (µm)*	*Endplate band width (µm)*	*%DW*
**E16**	69,138	11,227	16,238	67,310	24,790	36,830
**E18**	121,971	11,481	9,413	108,611	39,116	36,015

*Mean %DW values for WT and γ/ε-fc embryos*
	Wild Type	γ/ε-fc
**E16**	15,28±0,94	34,35±1,52
**E18**	12,36±0,72	32,59±3,04

Lack of AChR activity not only changes endplate distribution, but severely disrupts the innervation pattern, as also reported in *ChAT^−/−^* mice [12], [14]. The *γ/ε-fc* mutant displays a chaotic network of neurites increasing in complexity throughout late embryonic stages and leading to complete loss of directed axonal growth by E18 (Supplementary Figures S2, S3, S4).

### Scattered endplates are innervated

**Figure 5:**
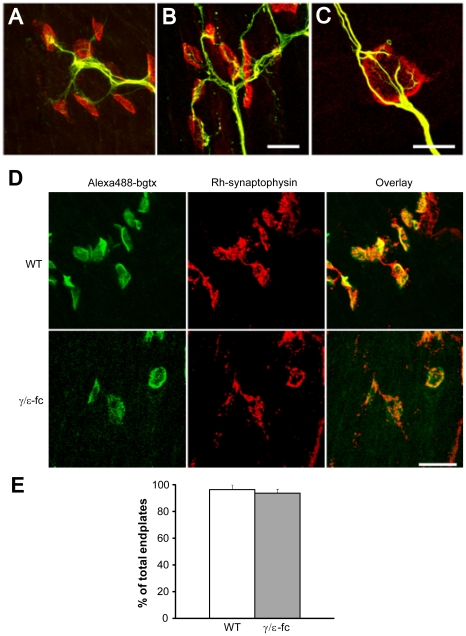
Innervation of individual endplates. (A) Neurofilament (green) and r-bgt (red) staining of nerve terminals and postsynaptic endplates in WT and (B) *γ/ε-fc* diaphragms of E18 embryos (scale bar: 20 µm). (C) Close-up of a single *γ/ε-fc* endplate, showing additional nerve sprouting after endplate innervation (scale bar: 10 µm). (D) Synaptophysin (Rh-synaptophysin, red) and Alexa-488-labeled α-bungarotoxin (green) staining of nerve terminals and postsynaptic endplates inWT and *γ/ε-fc* E18 embryos, showing co-localization of nerve terminals and endplates. Scale bar: 20 µm. (E) Percentage of synaptophysin-rich innervated endplates in WT (white) and *γ/ε-fc* (gray) embryos. n = 100 endplates, 2 embryos per point. All data are represented as mean ± SEM.

Next, we analyzed whether the presumptive endplates in *γ/ε-fc* embryos are properly innervated, despite the aberrant distribution and innervation pattern. In WT diaphragms, short nerve branches terminate on r-bgt stained endplates (Fig. 5A). Nerves in the *γ/ε-fc* diaphragm need longer branches to contact wider spread out endplates. In most cases they do not stop but continue to grow beyond the endplate, contacting additional endplates (Fig. 5B, C). The disorganization of this innervation pattern suggests that aberrantly positioned endplates attract nerves otherwise spreading directionless.

To find out whether presynaptic specializations are induced at the putative synaptic sites we tested for α-synaptophysin, which identifies presynaptic specializations at putative synaptic sites co-localizing with AChR clusters (Fig. 5D). We found no significant difference in the percentage of endplates contacted by nerve branches containing accumulations of α-synaptophysin between WT and *γ/ε-fc* embryos (96,42±3,57 for WT, 93,93±3,03 for *γ/ε-fc*, p>0,6) (Fig. 5E).

### Lack of AChR activity does not cause muscle atrophy

*In vitro* studies [18], [19] suggested that ACh-induced currents promote myogenesis and myotube survival [20]. Support for these activity-dependent roles of AChR derives from *in vivo* studies of mouse models lacking synaptic organizers such as agrin or MuSK, showing apparently normal myofiber growth and survival [4], [7]. In contrast, mice lacking ACh release and synaptic activity show widespread myofiber atrophy and degeneration of existing myotubes [13], [14].

**Figure 6:**
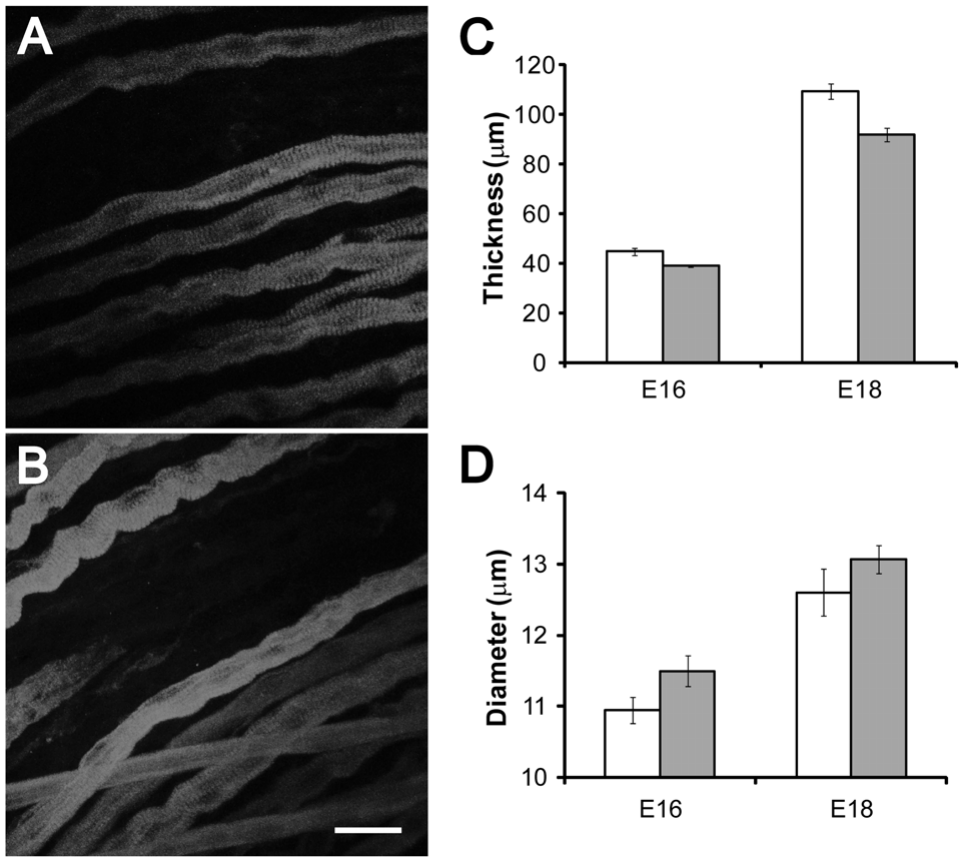
Muscle fiber diameter and diaphragm thickness. (A) Slow muscle fiber staining of the diaphragm of an E18 wild type and (B) *γ/ε-fc* embryo (scale bar: 25 µm). (C) Average diaphragm thickness of E16 and E18 WT (white) and *γ/ε-fc* (gray) embryos; n is 3 embryos for each bar. (D) Average muscle fiber diameter of slow muscle fibers in E16 and E18 WT (white) and *γ/ε-fc* (gray) embryos; n = 30 muscle fibers isolated from 3 separate embryos. Each bar represents the mean ± SEM.

To assess whether ACh-mediated regulation of myofiber growth and survival is activity-dependent, we analyzed diaphragms of WT and *γ/ε-fc* mice (Fig. 6A–D). Diaphragms of E16 and E18 *γ/ε-fc* embryos are only 15% thinner than those of their WT littermates (Fig. 6C) and herniation of the liver was undetectable in any *γ/ε-fc* homozygous embryo, unlike *ChAT^−/−^* embryos [14]. Since the size of individual myofibers could be reduced, we measured myofiber diameter in each diaphragm (Fig. 6D), focusing on slow muscle fibers, which comprise the first wave of myogenesis [21]. The diameters of *γ/ε-fc* and WT myofibers at E16 (10,95±0,19 for WT, 11,50±0,22 for *γ/ε-fc*, p = 0,061) and E18 (12,60±0,33 for WT, 13,07±0,20 for *γ/ε-fc*, p = 0,28) are similar suggesting that the absence of AChR-mediated postsynaptic activity does not reduce primary fiber growth during embryonic development, in contrast to changes occurring after postnatal muscle paralysis [22]. The small decrease in diaphragm thickness could result from reduced numbers of secondary muscle fibers. The results suggest that ACh mediates a protective effect on myofibers that is independent of postsynaptic activity.

### Muscle fibers present multiple endplates in the diaphragms of *γ/ε-fc* embryos

**Figure 7:**
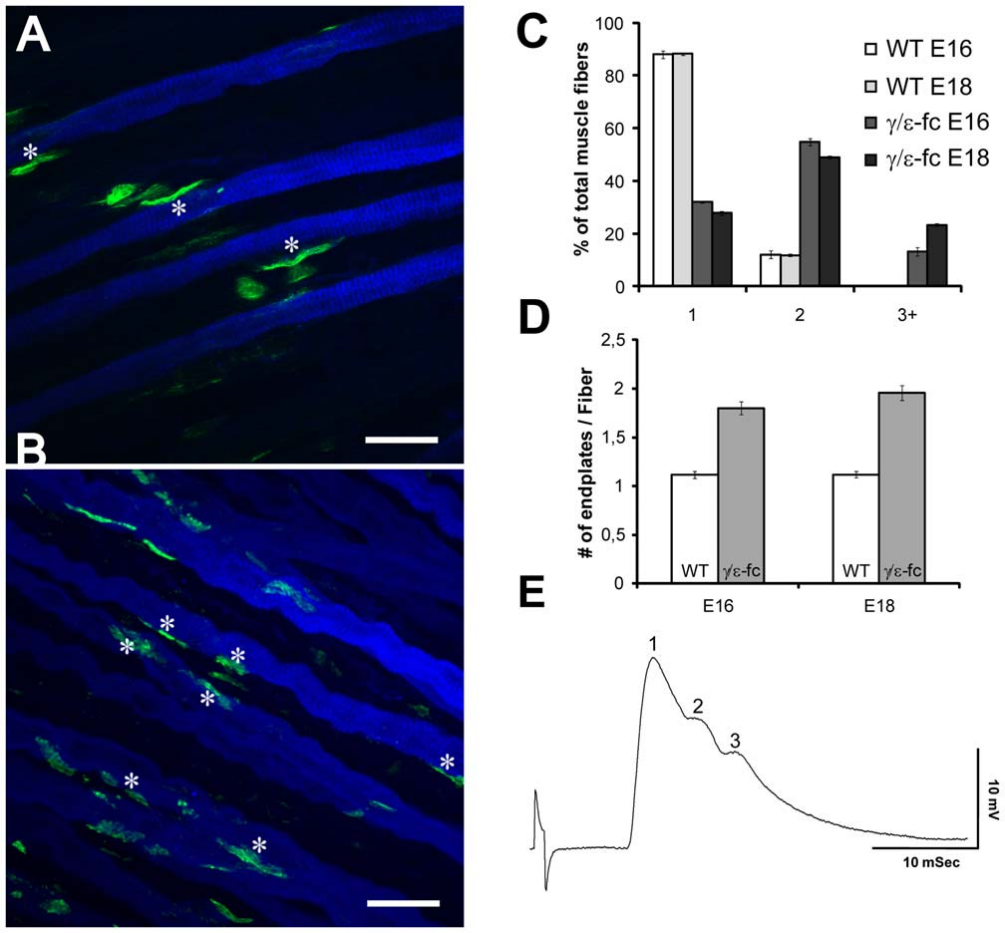
Presence of Multiple Endplates. (A) Slow myosin (blue) and Alexa 488-labeled α-bungarotoxin (green) staining of E16 WT and (B) *γ/ε-fc* diaphragm. The asterisks mark the location of endplates on individual muscle fibers. The *γ/ε-fc* diaphragm presents multiple endplates on individual muscle fibers (scale bar: 25 µm). (C) Percentages of analyzed muscle fibers of WT and *γ/ε-fc* embryos displaying one, two, or three or more endplates at E16 and E18. (D) Average number of endplates per muscle fiber in E18 wild type (1,12±0,03, white) and *γ/ε-fc* (1,95±0,07, gray) embryos (n = 30 muscle fibers, 3 embryos per point, p<0,001). All data are represented as mean ± SEM. (E) Electrophysiological evidence of polyinnervations of an endplate in the diaphragm muscle of an of an E19 *γ/ε-fc* embryo. The endplate potential consists of 3 separate components, which had the same stimulus threshold for initiation.

Aneural AChR clusters existing before innervation are destabilized by synaptogenesis, presumably due to the dispersal effect of ACh [8], [23]. This prevents formation of additional NMJs on the same myofiber through unknown, possibly activity-regulated retrograde signals. If lack of activity impairs retrograde signaling required to prevent the formation of multiple synapses on the same myofiber, fibers could present several fully innervated endplates. To test this hypothesis, we focused on slow muscle fibers, which are present in smaller numbers than fast fibers in the developing diaphragm and inspected individual fibers to detect multiple putative synaptic sites. Confocal images of endplates located on slow muscle fibers are shown in Fig. 7A, B. Evaluation (see Material and Methods) of 30 individual muscle fibers from each of 3 diaphragms of E16 and E18 WT and *γ/ε-fc* mice revealed that most E16 WT myofibers (∼90%) present only one endplate, with a small number presenting multiple clusters. The number of WT fibers with multiple endplates decreases between E16 and E18. In contrast, only 35% of E16 *γ/ε-fc* slow fibers display one endplate. Slow muscle fibers with two and three or more endplate sites made up approximately 55% and 10%, respectively, of the analyzed E16 *γ/ε-fc* myofibers. A very small number of *γ/ε-fc* fibers display up to five different endplate-like clusters. For E18 *γ/ε-fc* embryos, the number of slow fibers presenting one cluster decreases (25%), while the number of fibers presenting three or more clusters increases to 25% of all analyzed muscle fibers (Fig. 7D). These observations support the hypothesis that in *γ/ε-fc*, AChR cluster formation and innervation continue throughout development, regardless of already existing clusters. The percentage of fibers with multiple clusters (65% at E16, and 75% at E18) in *γ/ε-fc* mice is significantly higher than in *ChAT^−/−^* mice (50% at E17) [14]. The *γ/ε-fc* clusters appear contacted by synaptophysin-labeled nerve endings, suggesting that all detected clusters are innervated and represent synaptic sites.

Even though spontaneous transmitter release was rare, endplate potentials (epps) could occasionally be detected for E19 *γ/ε-fc* endplates. Fig. 7E presents one of these rare epps having multiple peaks. These multiple peaks indicate transmitter release from more than one terminal branch, suggesting that multiple endings innervating a single muscle fiber are functional.

## Discussion

Studies of genetically engineered mouse models suggest that nerve-independent prepatterning of AChRs requires MuSK, initiates postsynaptic differentiation [1], [3], [24], and appears to be primarily controlled by muscle [25]. On the other hand, it was suggested that NMJs can form in the absence of prepatterned AChRs, and NMJs are limited to spatially restricted regions responsive to neuronal factors such as agrin [26], [27]. *Zebrafish* studies indicate that MuSK signalling coordinates AChR prepatterning and axonal guidance. However, additional factors may restrict synapses to central muscle zones [28], [29]. Finally, it is unclear whether AChRs can themselves signal for purposes of synapse formation and/or guidance of growing motor nerves. Our experiments highlight the importance of AChR-mediated signals, and the *γ/ε-fc* mouse line demonstrates the specific contribution of postsynaptic activity to NMJ development.

AChRγ/ε-fcs are correctly assembled and clustered in endplates similar as AChRγ in WT mice. They are, however, functionally quiescent as indicated by the lack of mepp-exhibiting fibers. The few fibers displaying spontaneous endplate activity presented an abnormally low frequency, their mepps were reduced in amplitude and decayed faster than in WT myofibers. In fact, the *γ/ε-fc* 90% to 10% mepp decay time was similar to the value of 4.4±0.2 mSec recorded for adult WT muscle [30]. This briefer mepp duration is attributed to low expression and insertion of normal adult AChRε since mepps were also significantly smaller than for age matched WT endplates.

The wider distribution of endplates in *γ/ε-fc* mice is also observed for muscles with impaired activity due to ACh absence [14], [31]. It shows that AChR-mediated postsynaptic activity is crucial for the correct positioning of the central endplate band, supporting previous observations in mice expressing AChRε rather than AChRγ during embryonic development [32]. Muscle fibers in *γ/ε-fc* mice often contained several endplate-like specializations. These observations suggest that lack of ACh-evoked activity promotes increased AChR clustering beyond the “central nerve-responsive zone“ of WT mice.

In *γ/ε-fc* diaphragms, as in *ChAT^−/−^* diaphragms [12], [14], nerve branching is vastly increased. Nerve branching is directionless and rather than generating “neural” AChR clusters, the neurites appear arbitrarily attracted by widely distributed, presumably “aneural” AChR clusters. This implies that postsynaptic activity is unnecessary for axons to recognize sufficiently sized AChR clusters and to stabilize them. Activity, however, is necessary to trigger retrograde signals halting further neurite growth. In addition, an activity-independent effect of ACh might act as a dispersal factor at the single endplate level, thus preventing incorporation of excessive numbers of AChRs into individual endplates.

Compared to WT diaphragms, *γ/ε-fc* endplates display increased area as also observed for *ChAT^−/−^* endplates [12], [14], but also decreased AChR density. Lack of postsynaptic activity appears to induce irregular shapes in *γ/ε-fc* endplates, partially sustained by muscle-autonomous differentiation [33]. Thus, inactivity due to either ACh absence or silent AChRs might lead to different aberrations in endplate distribution and maturation. In contrast to the *ChAT^−/−^* phenotype [14], however, *γ/ε-fc* endplates did not exhibit precocious maturation at late developmental stages. Furthermore, *γ/ε-fc* endplate bands grow wider throughout prenatal development rather than remaining constant in size as for *ChAT^−/−^* diaphragms. This suggests that ACh release, even if unable to evoke functional electrophysiological responses, contributes to regulating the appearance of new AChR-rich sites on growing myofibers. Endplate size in *γ/ε-fc* animals is smaller than in *ChAT^−/−^* animals and total AChR number is smaller (as inferred from similar receptor densities but significantly different areas), adding to the evidence of ACh as a dispersal factor. Overall, the differences in endplate appearance between *γ/ε-fc* and *ChAT^−/−^* animals [14] can be attributed to the presence or absence of ACh, respectively. This interpretation suggests that ACh negatively regulates endplate differentiation.

**Figure 8:**
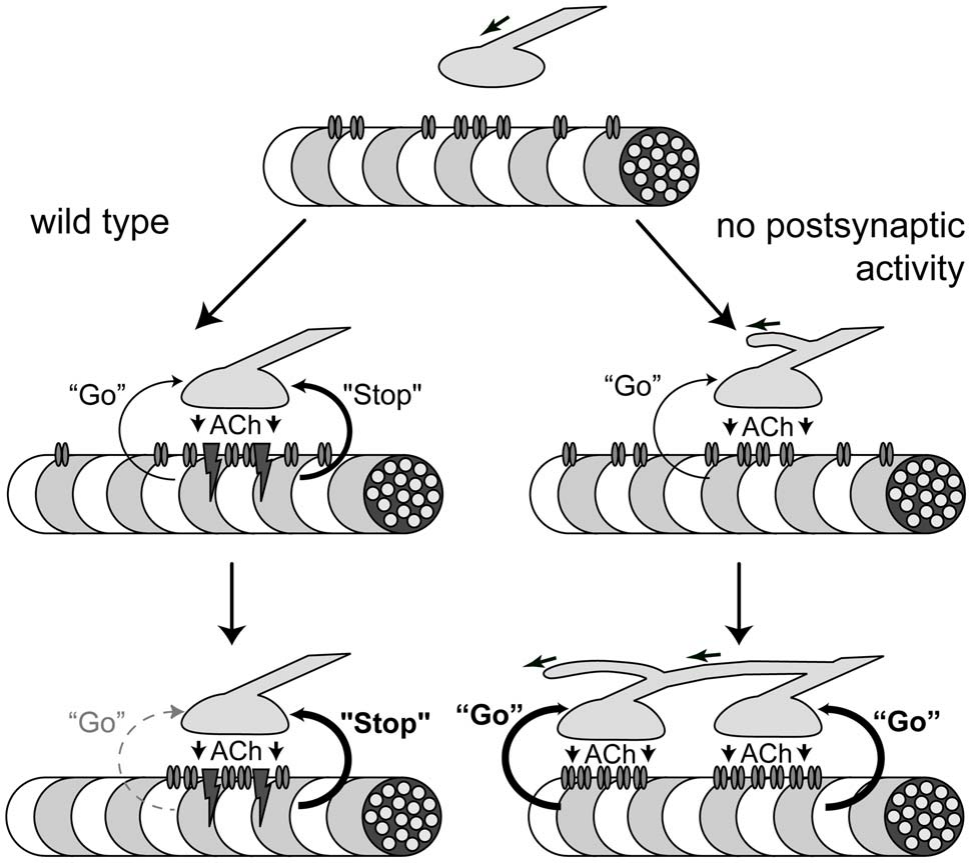
Retrograde Signaling at the NMJ. Model for retrograde signaling at the NMJ in wild type and mutant mice. ACh release from the presynaptic nerve terminal leads to a retrograde signal from muscle in the form of an activity-independent “go” signal promoting growth of the neurite and an activity-dependent “stop” signal halting neurite growth. The “stop” signal is dominant over the “go” signal. In wild type embryos the “stop” signal prevents further growth of the neurite once an endplate is formed. In the absence of postsynaptic activity, the “stop” signal does not occur and the activity-independent “go” signal triggers continuous neurite growth with the formation of additional endplates on the same muscle fiber.

In Fig. 8 we propose a speculative model in which ACh acts as a dispersal factor through two separate pathways. ACh exerts a global, myotube-level dispersal effect on AChR clusters through an activity-dependent pathway. This prevents extrasynaptic cluster formation and promotes existing AChR cluster dispersal [1], [7], [8]. Conversely, ACh acts on individual endplates through an activity-independent pathway, preventing excessive AChR clustering within an existing endplate.

In *γ/ε-fc* mice, the absence of activity-dependent dispersal allows formation of multiple clusters on individual myofibers. However, the presence of an activity-independent pathway prevents excessive clustering within individual endplates, thus increasing the number of endplates per myofiber. We also hypothesize that the activity-independent pathway prevents myotube degeneration and necrosis.

In *ChAT^−/−^* or *vAChT^−/−^* mice, both AChR pathways are absent (Misgeld et al. 2002; de Castro et al. 2009). While multiple receptor clusters can form on individual myofibers, absence of an activity-independent pathway may preferentially integrate newly synthesized AChRs into existing endplates, lowering the incidence of multiple clusters relative to the *γ/ε-fc* mice. Absence of an activity-independent pathway could also explain the apparent discrepancy between normal muscle appearance in *γ/ε-fc* embryos and atrophy and apoptosis detected in *ChAT^−/−^* or *vAChT^−/−^* animals.

Both AChR-mediated pathways could initiate retrograde signals altering nerve growth. The activity-dependent pathway appears to induce a dominant “stop” signal, suppressing further neurite growth [34]. This signal may be essential to development of a single NMJ as well as preventing nerve terminal sprouting. Conversely, the activity-independent pathway, normally masked by the dominant “stop” signal, may release a “go” signal to positively regulate neurite growth and branching.

In the *γ/ε-fc* mice, the “stop” signal is silenced. Therefore the unmasked, activity-independent “go” signal stimulates neurite growth, presumably attempting to form another functioning NMJ. By this process multiple contacts are established causing continuous widening of the *γ/ε-fc* endplate distribution throughout development.

In the absence of ACh release, both “stop” and “go” signals are missing, which may explain the absence of continuous endplate band widening in *ChAT^−/−^* embryos [14]. Given the differences between mouse lines, the activity-independent pathway might be mediated by ACh interacting with the α/δ binding site, which is intact in the *γ/ε-fc* line; it has been demonstrated that the two AChR binding sites differentially influence gating dynamics [35]. Furthermore, α/δ ACh binding could affect AChR-rapsyn interactions [36]. The latter interaction might represent the activity-independent AChR pathway.

Characterization of *ChAT^−/−^* [14] and *vAChT^−/−^* embryos [13] indicated the presence of muscle atrophy and apoptosis. Conversely, myofibers in *γ/ε-fc* diaphragms are similar to WT myofibers, suggesting that myotube growth was unaffected by lack of AChR-evoked postsynaptic activity. This agrees with data reported for *MuSK^−/−^* and *agrin^−/−^* embryos [4], [7]. Our data support a regulatory role of ACh in myogenesis [18], [19] and suggest that ACh myogenic activity does not occur through AChR-mediated postsynaptic activity. Furthermore, comparing data from *γ/ε-fc* embryos, *ChAT^−/−^* embryos [14] and denervated muscles [20] reveals profound differences in myotube growth and survival. These differences further support our hypothesis that ACh plays an important role in NMJ formation, partially independent of AChR-evoked postsynaptic activity.

The *γ/ε-fc* NMJ is very similar to the WT NMJ, containing the complete synaptic molecular machinery, including ACh. Thus, the *γ/ε-fc* mouse is an extremely powerful tool to discriminate between activity-dependent and -independent mechanisms essential to NMJ formation. Since different AChR subtypes are present in the central nervous system, activity-independent pathways downstream of ACh release might also exist there. These pathways might refine the current model for development of cholinergic synapses, and move towards a new understanding of the metabotropic roles of other ionotropic neurotransmitter receptors.

An important question which the *γ/ε-fc* mouse will help answer concerns the activity threshold that endplates require for stabilization and postnatal survival. Recent studies suggested that NMJs have an extremely high redundancy factor [16]. Another challenge involves the identification of specific, activity-induced signals in the postsynaptic region, which until now could not be separated from activity-independent signals. Finally, studies to rescue *γ/ε-fc* embryos from perinatal death will enable determination of which morphological changes are retained throughout adulthood despite the postnatal switch between AChRγ and AChRε.

## Materials and Methods

### Generation of the *γ/ε-fc* mouse line

A mouse ε cDNA fragment starting 5′at the XbaI site located in exon 4 to the 3′end including the stop codon was cloned into a 7-kb fragment of genomic γ subunit DNA sequence including native promoter sequences. The AChRγ/ε-fc subunit is schematically detailed in Fig. 1A. The XbaI site in exon 4 of the γ DNA was generated by silent mutation. The cDNA for the mouse ε subunit included the point mutation (C441T) replacing P121 with leucine (P121L). Humanized GFP was excised from a rat ε cDNA fusion construct via NheI/XcmI and cloned into the targeting construct. This ε-GFP subunit fragment contained no changes in functionally important sequences [37]. It was used because green fluorescent ε-GFP subunits were correctly assembled into AChRs with WT-like properties, that were targeted to endplates and clustered as fluorescent receptors [38].

A neomycin resistance cassette for selection purposes was added 3′ of the stop codon of the ε subunit cDNA. pBKS was used as an expression vector. The final targeting vector γ/εP121L-GFP-p1002 is shown schematically in Fig. 1B.

Embryonic stem (ES) cells [39] were used for gene targeting. Cells were selected with G418® (335–350 µg/ml). Correctly targeted clones were identified via PCR and Southern blot analysis conducted on HindIII-digested genomic DNA. ES cells from one correctly targeted clone were injected in blastocysts derived from C57Bl/6 mice (performed at ZTL, Heidelberg, Germany). Male chimeric mice were bred with C57Bl/6 females. Heterozygous offspring was used to obtain homozygous embryos that die at birth. Correct gene targeting in chimeric founders was confirmed by Southern blot analysis and DNA sequencing. Embryos were harvested after pregnant animals were sacrificed by CO_2_. They were immediately transferred in Ringer's solution (135 mM NaCl, 5.4 mM KCl, 1 mM MgCl_2_, 1.8 mM CaCl_2_, 5 mM Hepes) for further analysis. All animal experiments were carried out in accordance with the Guide for the Care and Use of Laboratory Animals published by the U.S. National Institute of Health (NIH Publication No. 85-23, revised 1996) and the European Community guidelines for the use of experimental animals. Use and care of animals followed German national laws and was approved by German authorities (TierSchG §§7; IBF Universität Heidelberg MPI/T-13/08).

### Genotyping of Animals

Genotyping was performed using Qiagen QIAamp DNA Mini Kit (Qiagen, Hilden, Germany). Primers used to identify the γ/ε-fc subunit and WT alleles were mg187 F (forward) GATGCGAAACTACGACCCC and mg536R (reverse) AGGAGGAGCGGAAGATGG for the wild type allele; mg187 F (forward) and mg785R (reverse) CAGAAATGAGCACGCAAGG for the AChRγ/ε-fc subunit. PCR conditions were 1 cycle 95°C for 15 minutes; 32 cycles 94°C for 45 s, 60°C for 45 s, 72°C for 1 m 30 s; followed by one cycle 72°C for 10 minutes. Southern blot hybridization of genomic DNA was performed using fluorescein-labeled probes (ECL Labeling Kit, Roche).

### Histological Analysis, Immunohistochemistry

Diaphragms were dissected and prepared as previously described [38]. AChRs were visualized with tetramethylrhodamine-, Alexa488- or FITC-labeled α-bungarotoxin at 1 µg/ml in PBS (Molecular Probes, Leiden, The Netherlands). Immunohistochemical staining of diaphragms has been described previously [16]. Anti-neurofilament antibodies (Chemicon, Temecula, U.S.A.) were used at a 1∶500 dilution, anti-synaptophysin (Zymed, San Francisco, U.S.A.) at a 1∶50 dilution, anti-slow myosin (Sigma, St. Louis, U.S.A.) at a 1∶5000 dilution.

### Endplate Localization and Estimation of Endplate Band Width

Acetylcholinesterase staining was performed according to Koelle and Friedenwald (1949). Endplates were imaged using a Zeiss Axioplan2 microscope (Carl Zeiss Inc.) and analyzed using ImageJ software (NIH, U.S.A.) with standard plug-ins. Lines were drawn from the inner to the outer edge of 6 different diaphragm regions and the distance of the first and last endplate on the line was measured starting from the inner edge of the diaphragm. At least 3 to 6 different lines in each region were evaluated. Data were analyzed in IgorPro (WaveMetrics, Lake Oswego, U.S.A.). Due to diaphragm size differences, bias was removed by presenting endplate band width as percentage of overall diaphragm width for each analyzed line. Standard error of the mean was calculated for each data series. Student's *t* tests were performed for statistical analysis, and a probability value (P) equal to or less than 0.05 was taken as criterion for statistical significance.

### Endplate Morphology

Images of endplates were taken using a Leica TCS NT (Leica Microsystems, Wetzlar, Germany) confocal microscope at 63× magnification, and fluorescent intensity as a measure of AChR density was evaluated as described previously [16].

Differential distribution of GFP- and r-bgt-labeled AChR across *γ/ε-fc* endplates was analyzed essentially as described by Yampolsky et al. (2008) and images were processed in ImageJ (NIH) using standard plug-ins. Maximum projections of en face views of endplates were taken, and five neighboring lateral axes were used to measure fluorescence intensities at every point and to obtain average values along these axes. The ratios of average green/red and red/green fluorescence intensities reflect directly the differential distribution of GFP-containing embryonic receptors versus non-GFP-tagged AChRε.

Circularity measurements were collected using ImageJ standard tools and data were analyzed in IgorPro (WaveMetrics, Lake Oswego, U.S.A.).Standard error of the mean was calculated for each data series. Student's *t* tests were performed for statistical analysis, and a probability value (P) equal to or less than 0,05 was taken as criterion for statistical significance.

Area measurements were collected using ImageJ standard tools, seelcting endplates arranged perpendicularly in regards to the light beam, in order to reduce the risk of artifacts. Data were analyzed in IgorPro (WaveMetrics, Lake Oswego, U.S.A.). Standard error of the mean was calculated for each data series. Student's *t* tests were performed for statistical analysis, and a probability value (P) equal to or less than 0,05 was taken as criterion for statistical significance.

### Measurement of Muscle Fiber Diameter and Diaphragm Thickness

Slow muscle fibers were stained with anti-slow myosin antibody. Stacks of confocal pictures were taken using a Leica SP2 (Leica Microsystems, Wetzlar, Germany) confocal microscope, and analyzed using ImageJ (NIH, U.S.A.) as well as Leica LAS AF Lite (Leica Microsystems, Wetzlar, Germany) software. For diaphragm thickness, three measurements were taken in different areas of the diaphragm by recording beginning level and end level of the diaphragm through optical analysis of confocal stacks. For muscle fiber diameter, horizontal and vertical diameters of muscle fibers from different regions of the diaphragm were measured in three separate locations and averaged to yield mean muscle fiber diameter. Data were analyzed in IgorPro (WaveMetrics, Lake Oswego, U.S.A.). Standard error of the mean was calculated for each data series. Student's *t* tests were performed for statistical analysis, and a probability value (P) equal to or less than 0,05 was taken as criterion for statistical significance.

### Count of Endplates per Muscle Fiber

Confocal pictures were taken using a Leica TCS NT (Leica Microsystems) confocal microscope, and analyzed using ImageJ (NIH, U.S.A.) with Volume Viewer plug-in (K.U.Barthel, Internationale Medieniformatik, Berlin, Germany). Pseudo-3D reconstructions of each section were created using Volume Viewer and individual muscle fibers were traced from one end to the other. Endplates visualized with Alexa488-labeled α-bungarotoxin laying over antibody-stained muscle fibers were analyzed optically. Data were analyzed in IgorPro (WaveMetrics, Lake Oswego, U.S.A.). Standard error of the mean was calculated for each data series. Student's *t* tests were performed for statistical analysis, and a probability value (P) equal to or less than 0,05 was taken as criterion for statistical significance.

### Innervation Pattern

Nerves visualized with anti-neurofilament antibody staining were analyzed with ImageJ (NIH, U.S.A.) using NeuronJ plug-in (E. Meijering, Erasmus MC – University Medical Center Rotterdam, The Netherlands). Secondary neurites were traced starting at the point of divergence from the primary phrenic nerve trunk and up to their lateral distal ending. Numerical data were processed in IgorPro (WaveMetrics, Lake Oswego, U.S.A.). Neurite lengths were grouped into arrays of 1 µm each in order to assess length distribution within the neuronal population. Standard error of the mean was calculated for each data series. Student's *t* tests were performed for statistical analysis, and a probability value (P) equal to or less than 0.05 was taken as criterion for statistical significance.

### Motoneuron Survival

Embryonic tissue was fixated with zinc and embedded in paraffin. 7 µm thick sections of the embedded tissue were prepared and analyzed as described previously [16].

### Recording and Analysis of Miniature Endplate Potentials (mepps)

Miniature endplate potentials (mepps) were measured in diaphragms of E16 and E18 *γ/ε-fc* embryos. WT mice, as well as littermates of *γ/ε-fc* embryos expressing WT AChRγ provided control diaphragms. Embryos were harvested (see above) and placed into normal rat ringer containing (mM): NaCl (135), KCl (5.4), HEPES (5), MgCl_2_ (1), CaCl_2_, D-glucose (5.5). Tail segments of all embryos were frozen for genotyping. Phrenic nerve/diaphragm preparations were dissected for electrophysiological study. Each preparation was pinned to a Sylgard-lined Plexiglass chamber containing rat ringer (22°C) and viewed with an upright microscope. Muscle fibers were impaled with electrodes (15–25 MΩ, 3 M KCl) and membrane potentials were amplified (Axoclamp-2B, Axon Instruments, Foster City, CA,USA), digitized (Digidata 1200, Axon Instruments), acquired and analyzed with PCLAMP software (version 9.2, Axon Instruments). Records were acquired for at least 1 min to detect mepps. The percentage of impalements detecting mepps indicates the success of endplate detection. Because visual identification of endplates in embryonic muscles is difficult, electrophysiological studies were performed in a triple blind fashion. That is, separate investigators evaluated either mepps or embryo genotypes. The results were then independently compiled by a third investigator.

## Supporting Information

Figure S1 **Endplate morphology by diaphragm region.** (A) Area size (in µm^2^) of wild type (white) and *γ/ε-fc* (gray) endplates at E16 and E18, arranged by diaphragm region (sternal or lumbar). (B) Circularity of wild type (white) and *γ/ε-fc* (gray) endplates at E16 and E18, by diaphragm region; a value of 1.0 indicates a perfect circle. n = 60 endplates, 3 embryos per point. All error bars indicate SEM.
(TIF)

Figure S2 **Innervation Pattern.** (A) Composite pictures of left and right hemidiaphragms from an E16 (2 left panels) and a right hemidiaphragm of an E18 (right panel) wild type (WT) embryo. (B) Composite pictures of left and right hemidiaphragms from an E16 *γ/ε-fc* embryo (2 left panels) and a right hemidiaphragm from an E18 (right panel) *γ/ε-fc* embryo. See also Figure S3 and Figure S4.
(TIF)

Figure S3 **Innervation Pattern Analysis.** Analysis of average number of neurites and average neurite length in diaphragms of E16 and E18 wild type and *γ/ε-fc* embryos. Left hemidiaphragm: S1–2; right hemidiaphragm: S3–4. Wild type: white; *γ/ε-fc*: gray. (A) Average number of secondary and higher-order neurites in the left and right hemidiaphragm of wild type and *γ/ε-fc* embryos at E16. (B) Average length of secondary and higher-order neurites (in µm) in the left and right hemidiaphragm of wild type and *γ/ε-fc* embryos at E16. (C) Average number of secondary and higher-order neurites in the left and right hemidiaphragm of wild type and *γ/ε-fc* embryos at E18. (D) Average length of secondary and higher-order neurites (in µm) in the left and right hemidiaphragm of wild type and *γ/ε-fc* embryos at E18. (E) Comparison of the average number of secondary and higher-order neurites in diaphragms of wild type and *γ/ε-fc* embryos at E16 and E18. (F) Comparison of the average length of secondary and higher-order neurites (in µm) in diaphragms of wild type and *γ/ε-fc* embryos at E16 and E18. All error bars indicate SEM.
(TIF)

Figure S4 Motoneuron Survival. (A) ChAT staining in whole-mount paraffin section in the cervical region of a wild type E18 animal. Scale bar = 200 µm. (B) Comparison of total motoneuron numbers in wild type (white) and *γ/ε-fc* (gray) embryos at age E16 and E18. n = 10 spinal cord sections, 3 embryos per point. Error bars indicate SEM.
(TIF)

Table S1 **Occurrence and properties of mepps at WT and** ***γ/ε-fc*** **endplates.**
(DOC)
