# Supplementary figures and images for: Novel Mouse Model Reveals Distinct Activity-Dependent and –Independent Contributions to Synapse Development
Source: PLoS One. 2011 Jan 31;6(1):e16469. doi: 10.1371/journal.pone.0016469 (PMC3031568; doi:10.1371/journal.pone.0016469)

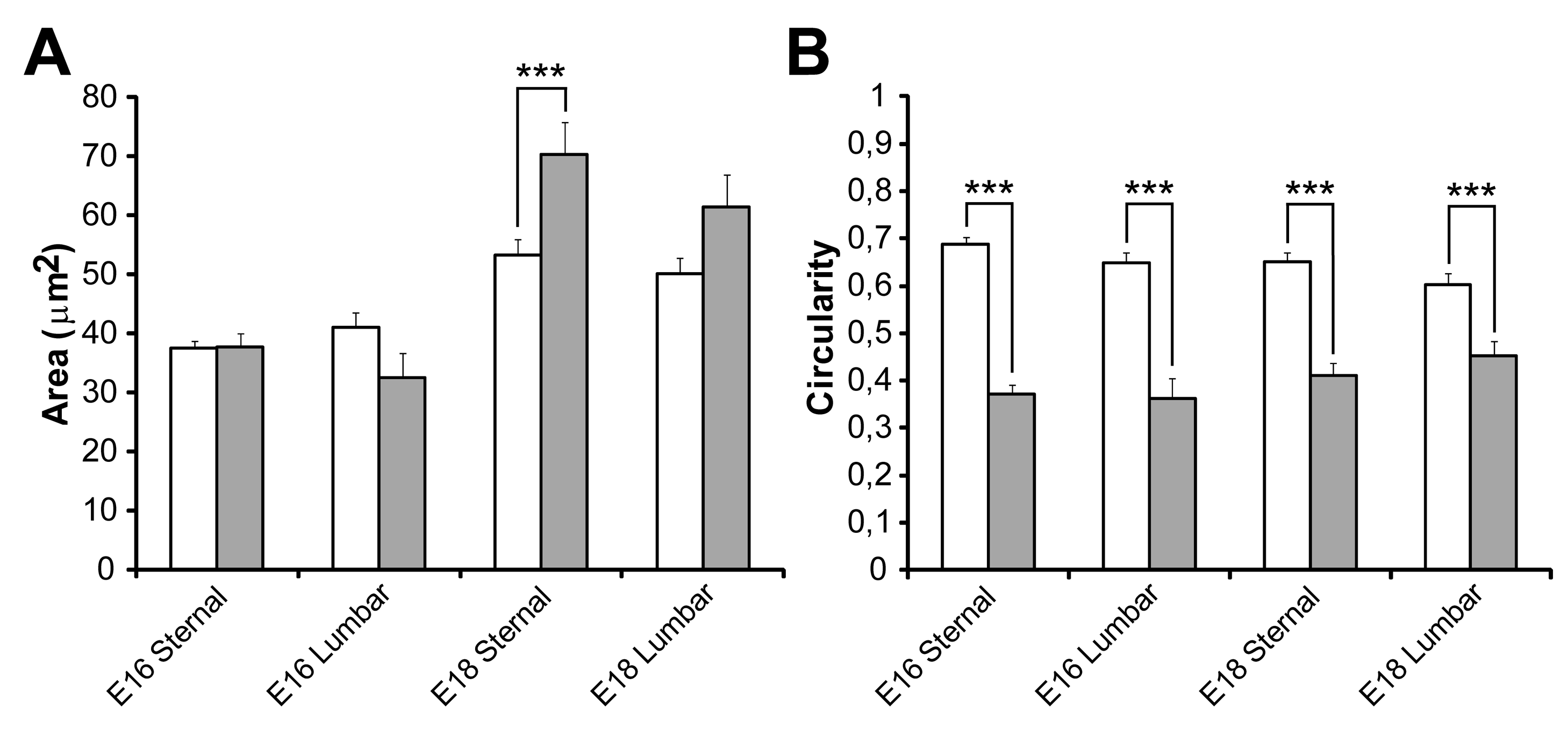

Supplement: Figure S1 — Endplate morphology by diaphragm region. (A) Area size (in µm2) of wild type (white) and γ/ε-fc (gray) endplates at E16 and E18, arranged by diaphragm region (sternal or lumbar). (B) Circularity of wild type (white) and γ/ε-fc (gray) endplates at E16 and E18, by diaphragm region; a value of 1.0 indicates a perfect circle. n = 60 endplates, 3 embryos per point. All error bars indicate SEM. (TIF) [file pone.0016469.s001.tif]

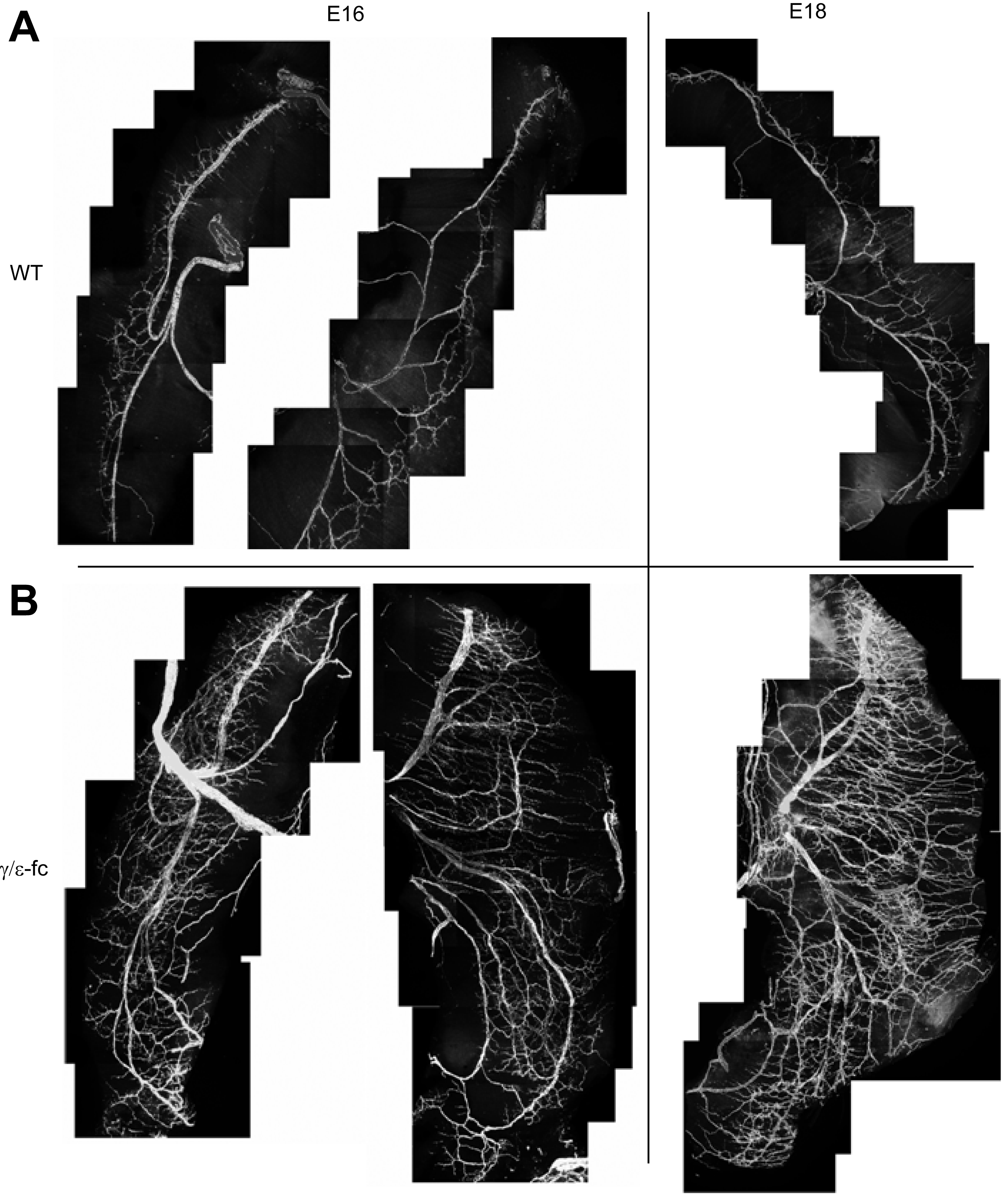

Supplement: Figure S2 — Innervation Pattern. (A) Composite pictures of left and right hemidiaphragms from an E16 (2 left panels) and a right hemidiaphragm of an E18 (right panel) wild type (WT) embryo. (B) Composite pictures of left and right hemidiaphragms from an E16 γ/ε-fc embryo (2 left panels) and a right hemidiaphragm from an E18 (right panel) γ/ε-fc embryo. See also Figure S3 and Figure S4. (TIF) [file pone.0016469.s002.tif]

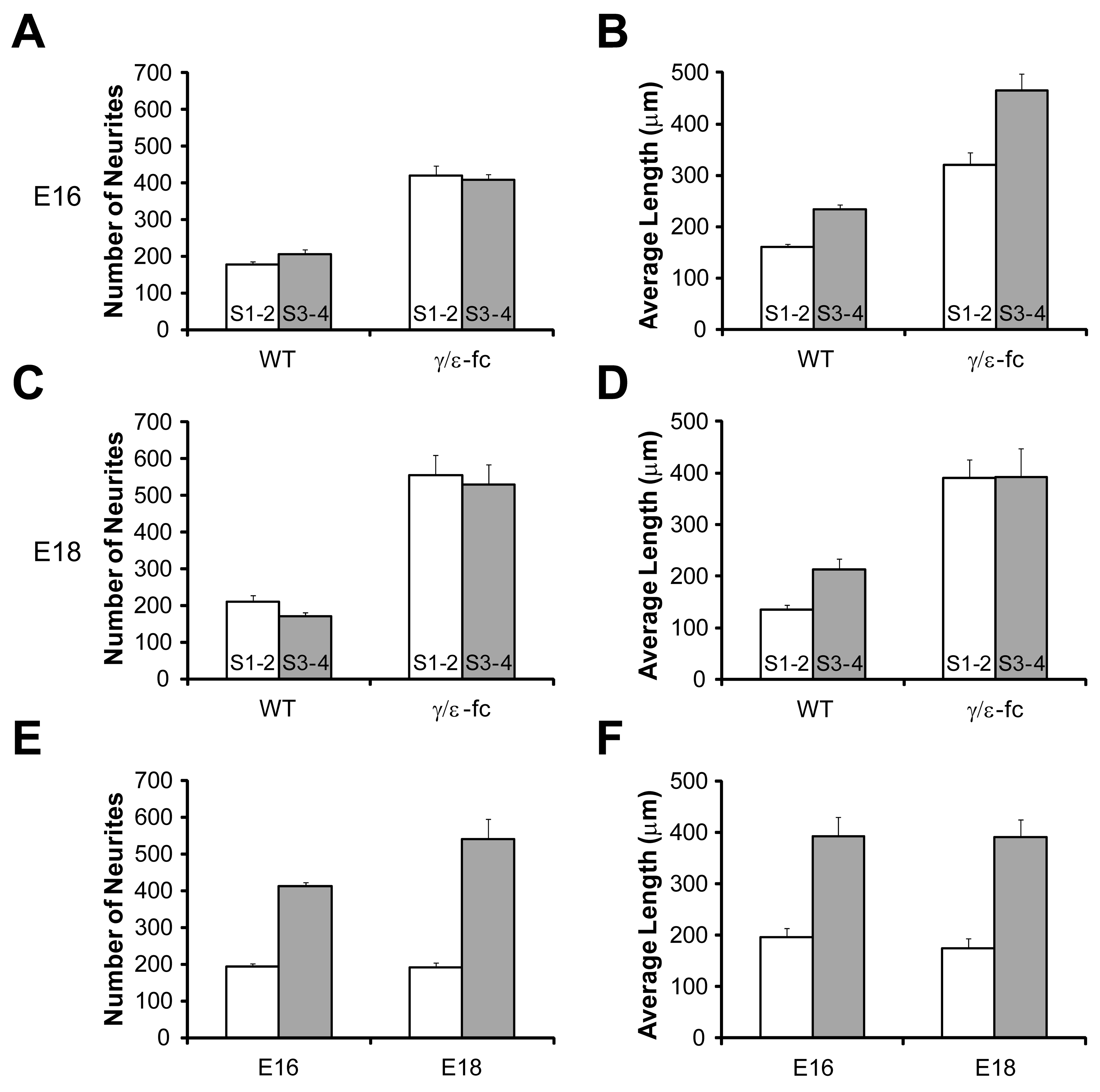

Supplement: Figure S3 — Innervation Pattern Analysis. Analysis of average number of neurites and average neurite length in diaphragms of E16 and E18 wild type and γ/ε-fc embryos. Left hemidiaphragm: S1–2; right hemidiaphragm: S3–4. Wild type: white; γ/ε-fc: gray. (A) Average number of secondary and higher-order neurites in the left and right hemidiaphragm of wild type and γ/ε-fc embryos at E16. (B) Average length of secondary and higher-order neurites (in µm) in the left and right hemidiaphragm of wild type and γ/ε-fc embryos at E16. (C) Average number of secondary and higher-order neurites in the left and right hemidiaphragm of wild type and γ/ε-fc embryos at E18. (D) Average length of secondary and higher-order neurites (in µm) in the left and right hemidiaphragm of wild type and γ/ε-fc embryos at E18. (E) Comparison of the average number of secondary and higher-order neurites in diaphragms of wild type and γ/ε-fc embryos at E16 and E18. (F) Comparison of the average length of secondary and higher-order neurites (in µm) in diaphragms of wild type and γ/ε-fc embryos at E16 and E18. All error bars indicate SEM. (TIF) [file pone.0016469.s003.tif]

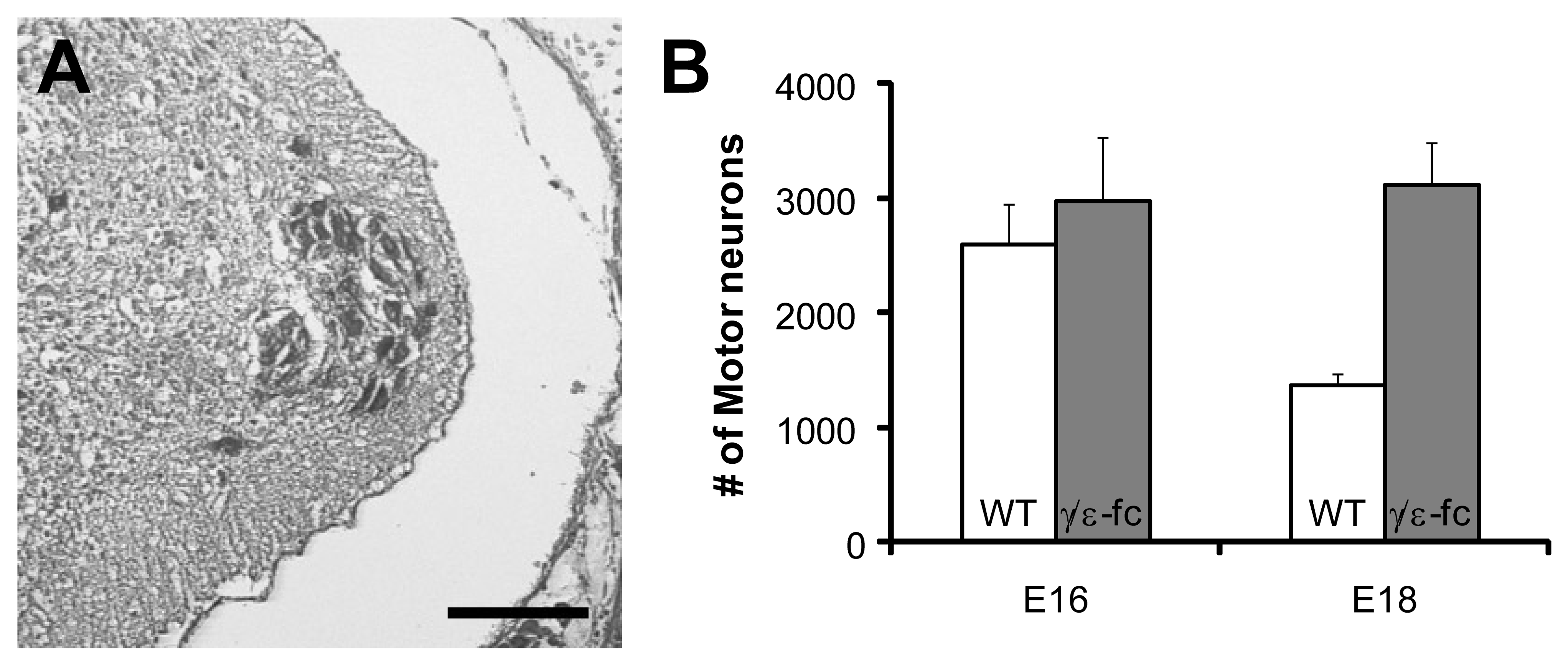

Supplement: Figure S4 — Motoneuron Survival. (A) ChAT staining in whole-mount paraffin section in the cervical region of a wild type E18 animal. Scale bar = 200 µm. (B) Comparison of total motoneuron numbers in wild type (white) and γ/ε-fc (gray) embryos at age E16 and E18. n = 10 spinal cord sections, 3 embryos per point. Error bars indicate SEM. (TIF) [file pone.0016469.s004.tif]
