# Supplementary material for: Novel Mouse Model Reveals Distinct Activity-Dependent and –Independent Contributions to Synapse Development
Source: PLoS One. 2011 Jan 31;6(1):e16469. doi: 10.1371/journal.pone.0016469 (PMC3031568; doi:10.1371/journal.pone.0016469)
Supplement: Table S1 — Occurrence and properties of mepps at WT and γ/ε-fc endplates. (DOC) [file pone.0016469.s005.doc]

| **Table SI.** Occurrence and properties of mepps at WT and g/e-fc endplates. | | | | | | |
| --- | --- | --- | --- | --- | --- | --- |
|  | | # of muscle fibers ^b^ | % of muscle fibers with mepps ^b^ | Mepp frequency (Sec^-1^) ^c^ | Mepp amplitude (mV) | 90% to 10% decay time (mSec) |
| E16 | WT  (4, 8) ^a^ | 86 | 45 % | 0,39 ± 0,20  (3, 37) ^d^ | 1.18 ± 0.03  (3, 37, 829) | 13.5 ± 0.46  (3, 37, 829) |
|  | γ/ε-fc  (2, 5) ^a^ | 83 | 4 % | 0,03 ± 0,01  (3, 3) ^d^ | 0.38 ± 0.08**^*^**  (3, 3, 6) | 7.3 ± 1.12**^*^**  (3, 3, 6) |
| E18 | WT  (5, 8) ^a^ | 148 | 64 % | 0,22 ± 0,06  (8, 94) ^d^ | 1.01 ± 0.06  (3, 47, 170) ^e^ | 25.0 ± 1.36  (3, 47, 170) ^e^ |
|  | γ/ε-fc  (5, 9) ^a^ | 171 | 26 % | 0,07 ± 0,02  (9, 45) ^d^ | 0.68 ± 0.04**^*^**  (5, 42,123) ^e^ | 4.72 ± 0.31**^*^**  (5, 42, 123) ^e^ |

^a^ Number of litters and number of embryos with this genotype. WT embryos included both littermates of the homozygous embryos, and embryos produced by WT matings.

^b^ Intracellular recording was maintained for a minimum of 1 minute to detect mepps in these muscle fibers.

^c^ Expressed as mean ± SEM.

^d^ Number of muscles and muscle fibers.

^e^ Number of muscles, muscle fibers and mepps.

**^*^** Difference between WT and γ/ε-fc means is statistically significant (p<0.01).
